# Pediatric Melatonin Ingestions — United States, 2012–2021

**DOI:** 10.15585/mmwr.mm7122a1

**Published:** 2022-06-03

**Authors:** Karima Lelak, Varun Vohra, Mark I. Neuman, Michael S. Toce, Usha Sethuraman

**Affiliations:** ^1^Department of Pediatrics, Children’s Hospital of Michigan, Detroit Michigan; ^2^Department of Emergency Medicine, Wayne State University School of Medicine, Detroit Michigan; ^3^Division of Emergency Medicine, Department of Pediatrics, Boston Children’s Hospital, Boston Massachusetts; ^4^Department of Pediatrics, Central Michigan University, Detroit Michigan.

Melatonin is an endogenous neurohormone that regulates the sleep-wake cycle (*1*). It is used therapeutically for insomnia in adults and for primary sleep disorders in children (*2*). Melatonin is regulated by the Food and Drug Administration (FDA) as a dietary supplement. Various synthetic melatonin preparations are widely available over the counter (OTC) in the United States with sales increasing from $285 million in 2016 to $821 million in 2020 (*3*). Children are at increased risk for melatonin exposure because of the supplement’s widespread use and growing popularity as a sleep aid. In 2020, melatonin became the most frequently ingested substance among children reported to national poison control centers (*4*); however, more research is needed to describe the toxicity and outcomes associated with melatonin ingestions in children. This study assessed isolated melatonin ingestions among the pediatric population (defined here as children, adolescents, and young adults aged ≤19 years) during January 1, 2012–December 31, 2021, using the American Association of Poison Control Centers’ National Poison Data System (NPDS). During the 10-year study period, 260,435 pediatric melatonin ingestions were reported to NPDS, and the annual number of ingestions increased 530%. In addition, pediatric melatonin ingestions accounted for 4.9% of all pediatric ingestions reported to poison control centers in 2021 compared with 0.6% in 2012. Pediatric hospitalizations and more serious outcomes due to melatonin ingestions increased during the study period, primarily related to an increase in unintentional ingestions among children aged ≤5 years. Five children required mechanical ventilation, and two died. Consumers and health care professionals should be encouraged to report any melatonin product–related adverse events to MedWatch, the FDA’s medical product safety reporting program. Public health initiatives should focus on raising awareness of increasing numbers of melatonin ingestions among children and on the development of preventive measures to eliminate this risk.

This was a cross-sectional study of pediatric melatonin ingestions reported to U.S. poison control centers. All closed cases of single substance melatonin ingestions (generic code 0201106) involving children, adolescents, and young adults aged ≤19 years during January 1, 2012–December 31, 2021, were included (*5*). A closed case is one for which the regional poison control center determined that either no further follow-up or recommendations were required or no further information on the case was available (*5*). Aggregate national data were abstracted from NPDS (*5*). Noningestion routes of exposure, information requests, exposures with unknown age, and nonhuman exposures were excluded. Abstracted data included age group (≤5, 6–12, and 13–19 years), sex, ingestion reason (unintentional versus intentional), exposure and management site, disposition, and medical outcome. Those managed on-site included children treated at home or any other non–health care site. Standard descriptive statistics were used to describe and compare variables of interest. Rates (exposures per 100,000 population aged ≤19 years) were calculated using population estimates from the U.S. Census Bureau (*6*). More serious outcomes were defined as a moderate or major effect or death, as defined by the NPDS Coding Manual (*5*). Moderate effects include symptoms following an exposure that are more pronounced or systemic in nature and warrant a treatment intervention but are not life-threatening. Major effects involve symptoms considered life-threatening or that result in substantial residual disability. This study was determined to be nonhuman research and was exempt from human subject review by the Institutional Review Board of Central Michigan University.*

During 2012–2021, a total of 260,435 pediatric melatonin ingestions were reported to poison control centers, representing 2.25% of all pediatric ingestions reported during the same period. The majority of ingestions were unintentional (94.3%), involved males aged ≤5 years, occurred in the home (99.0%), and were managed on-site (88.3%) (Table). Most children (82.8%) were asymptomatic. Among those with reported symptoms, most involved the gastrointestinal, cardiovascular, or central nervous systems. Among 27,795 patients who received care at a health care facility, 19,892 (71.6%) were discharged, 4,097 (14.7%) were hospitalized, and 287 (1.0%) required intensive care. Among all melatonin ingestions, 4,555 (1.6%) resulted in more serious outcomes. Five children required mechanical ventilation, and two died. Both deaths occurred in children aged <2 years (3 months and 13 months) and occurred in the home. One ingestion involved intentional medication misuse; the reason for the other is unknown.

**TABLE:** Demographics and clinical characteristics of pediatric melatonin ingestions reported to poison control centers (N = 260,435) — United States, 2012–2021

Characteristic	Ingestions, no. (%)
**Age group, yrs**	
≤5	218,136 (83.8)
6–12	28,606 (11.0)
13–19	13,693 (5.2)
**Sex**	
Male	141,301 (54.3)
Female	117,872 (45.2)
Unknown	1,262 (0.5)
**Reason for ingestion**	
Unintentional	245,596 (94.3)
Intentional	13,722 (5.3)
Other	1,117 (0.4)
**Exposure site**	
Residence	257,761 (99.0)
School	561 (0.2)
Other	2,113 (0.8)
**Clinical effects**	
Asymptomatic	219,770 (82.8)
Symptomatic	45,647 (17.2)
CNS	37,164 (81.4)
Gastrointestinal	4,655 (10.2)
Cardiovascular	1,147 (2.5)
Metabolic	346 (0.8)
Other	2,335 (5.1)
**Outcome**	
No effect*	78,423 (30.1)
Minor effect^†^	176,435 (67.8)
More serious outcomes^§^	3,211 (1.2)
Death	2
Other^¶^	2,366 (0.9)
**Management site**	
Managed on-site (non-HCF)	230,032 (88.3)
Managed at HCF	27,795 (10.7)
Unknown	2,608 (1.0)
**Disposition of patients managed at HCF** **(n = 27,795)**	
Hospitalized	4,097 (14.7)
ICU	287 (1.0)
Treated and released	19,892 (71.6)
Other	3,806 (13.7)

**Abbreviations**: CNS = central nervous system; HCF = health care facility; ICU = intensive care unit.

* No signs or symptoms.

^†^ Minimally bothersome symptoms, self-limited, and resolved without intervention (e.g., self-limited gastrointestinal symptoms).

^§^ More serious outcomes included moderate effect (systemic symptoms requiring intervention; not life-threatening [e.g., brief seizure readily resolved with treatment, or high fever]), major effect (life-threatening symptoms [e.g., status epilepticus or respiratory failure requiring intubation]), and death.

^¶^ Cases that were not followed or unable to be followed to a known outcome but judged as likely nontoxic exposures or exposure deemed not responsible to the effect.

The number of pediatric melatonin ingestions increased 530% from 8,337 in 2012 to 52,563 in 2021, with the largest yearly increase (37.9%) occurring from 2019 to 2020. In 2021, pediatric melatonin ingestions accounted for 4.9% of all pediatric ingestions compared with 0.6% in 2012. The annual rate of ingestions per 100,000 U.S. population increased during the 10-year study period (Figure 1). This resulted largely from an increase in unintentional ingestions among children aged ≤5 years. There was also an increase in the number of ingestions requiring hospitalization and in those resulting in more serious outcomes (Figure 2). Most hospitalized patients were teenagers with intentional ingestions, whereas the largest increase in hospitalization occurred among children aged ≤5 years with unintentional ingestions.

**FIGURE 1:**
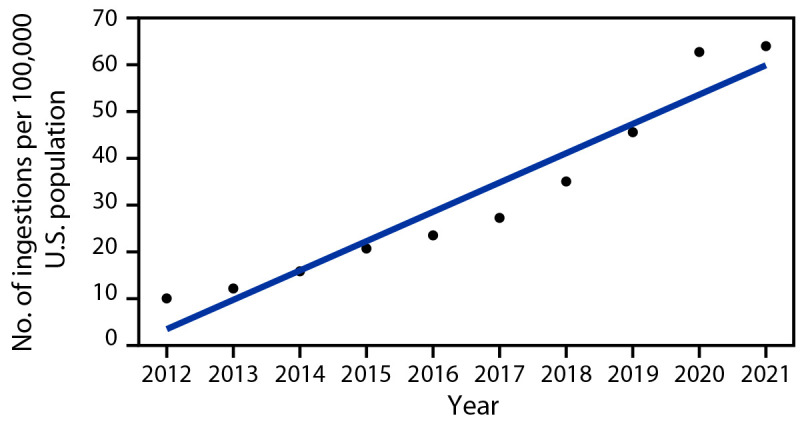
Rate* of pediatric^†^ melatonin ingestions reported to poison control centers, by year^§^ — United States, 2012–2021 * Ingestions per 100,000 population, based on U.S. Census Bureau Annual Estimate. ^†^ Aged ≤19 years. ^§^ Linear trend, p<0.001.

**FIGURE 2:**
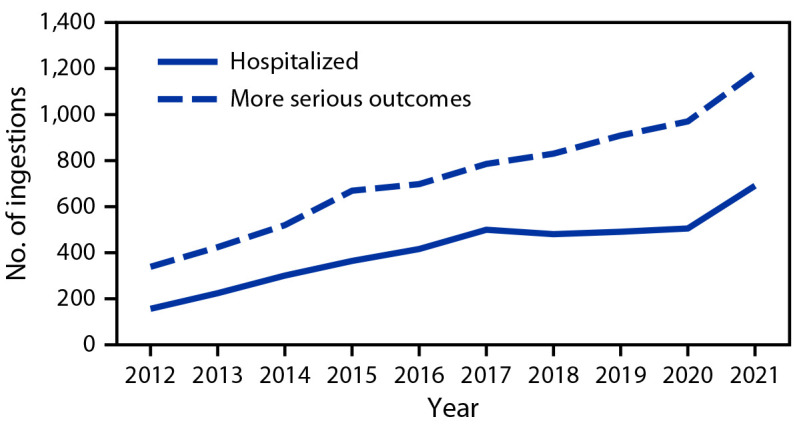
Number of pediatric* melatonin ingestions reported^†^ to poison control centers, by outcome and year — United States, 2012–2021 * Aged ≤19 years. ^†^ More serious outcomes include moderate or major effect or death, as defined by the National Poison Data System Coding Manual. Disposition (including hospitalization) and medical outcome (including more serious outcomes) are not mutually exclusive because persons with more serious outcomes are likely to be hospitalized.

## Discussion

Pediatric melatonin ingestions reported to U.S. poison control centers, including those requiring hospitalization and those with more serious outcomes, have increased during the past decade. Melatonin is widely available in tablet, capsule, liquid, and gummy formulations. It is cost-effective and offers an OTC therapeutic alternative to enhance sleep without use of potentially habit-forming sedative-hypnotics (*7*). Consequently, its use has increased in both adults and children (*7*,*8*). In addition, growth in the national melatonin market has occurred in response to public demand, with sales in the United States increasing by approximately 150% between 2016 and 2020 (*2*). Increased sales, availability, and widespread use have likely resulted in increased access and exposure risk among children in the home.

The largest annual increase in pediatric melatonin ingestions coincided with the onset of the COVID-19 pandemic. Unintentional ingestions were the primary drivers of this increase. This might be related to increased accessibility of melatonin during the pandemic, as children spent more time at home because of stay-at-home orders and school closures. Further, reports of increasing sleep disturbances during the pandemic might have led to increased availability of melatonin in the home (*9*). This pandemic-related increase in accessibility and availability might have contributed to increased exposures in children.

Hospitalizations and more serious outcomes due to melatonin ingestions have increased in children. Although reasons for this are unclear, one consideration is the variability in melatonin content across products (*10*). In addition, a previous study reported melatonin content not meeting label claims within a 10% margin in approximately 71% of supplements sold in Ontario, Canada (*10*). The same study reported significant sample variability (478%) along with melatonin content varying by as much as 465% between lots of the same product. The most variation was found in the chewable formulation, which is most likely to be used by children. In addition, serotonin, a breakdown product of melatonin, was found in 26% of supplements at potentially clinically significant doses that can increase the risk for serotonin toxicity in children (*10*). Quality control issues prompted a health legislation intervention banning the sale of OTC melatonin products in Canada. Similar drug quality studies and legislation initiatives in the United States are lacking. In the United States, melatonin is categorized as a dietary supplement, requires no prescription, and is subject to less regulatory oversight. Increasing use of OTC melatonin in various formulations, lack of robust manufacturing regulations, and varied dosing recommendations can place children at risk for potential adverse events. This report highlights the need for more research into the causes of increased melatonin ingestions among children and for public health initiatives to raise awareness. Child-resistant packaging for this supplement should be considered, and health care providers should warn parents about potential toxic consequences of melatonin exposure.

The findings in this report are subject to at least three limitations. First, poison control center data rely on passive, voluntary, and self-reported case communication that might underestimate actual exposures and lead to selection and information bias. Second, the American Association of Poison Control Centers is not able to confirm the accuracy of each case reported to poison control centers, and individual chart review of all cases could not be performed. Finally, poison control center data do not include patient medical records or medical examiner report, and confirmation of whether a death was secondary to toxic effects solely from melatonin or because of comorbidities was not possible.

Melatonin ingestions and related hospitalizations have increased in children during the past decade. The largest increase occurred during the COVID-19 pandemic. Health care providers should advise parents regarding the safe storage and appropriate use of melatonin. Further, consumers and health care professionals should be encouraged to report any melatonin product–related adverse events to MedWatch, the FDA’s medical product safety reporting program. These results might help guide health legislators regarding the need for public health measures to raise awareness of increasing pediatric melatonin ingestions and to develop preventative measures to eliminate this risk.

**Summary**

**What is already known about this topic?**

Melatonin is regulated by the Food and Drug Administration as a dietary supplement and is a widely available over-the-counter sleep aid for adults and children.

**What is added by this report?**

During 2012–2021, the annual number of pediatric ingestions of melatonin increased 530% with a total of 260,435 ingestions reported. Pediatric hospitalizations and more serious outcomes also increased, primarily because of an increase in unintentional melatonin ingestions in children aged ≤5 years.

**What are the implications for public health practice?**

Increasing use of over-the-counter melatonin might place children at risk for potential adverse events. Public health initiatives should focus on raising awareness of increasing melatonin ingestions among children and on preventive measures to eliminate this risk.

## Footnotes

* 45 C.F.R. part 46; 21 C.F.R. part 56.
