# *Erratum*: Vol. 71, No. 22

**DOI:** 10.15585/mmwr.mm7127a4

**Published:** 2022-07-08

In the report, “Pediatric Melatonin Ingestions — United States, 2012–2021,” on page 726 in the first full paragraph, the third sentence should have read, “Most children (**84.4%**) were asymptomatic.”

On page 727, the Table contained multiple errors: rows 18 and 19 (with the headings “Asymptomatic” and “Symptomatic”) should have been deleted, the final original footnote should have read, “**Cases confirmed as nonexposures and exposures deemed not responsible for the effect,**” two additional footnotes should have been included, and all footnotes should have been reordered. In addition, the abbreviation “**RCF = relative contribution to fatality**” should have been included. The Table has been updated accordingly.

**TABLE:** Demographics and clinical characteristics of pediatric melatonin ingestions reported to poison control centers (N = 260,435) — United States, 2012–2021

Characteristic	Ingestions, no. (%)
**Age group, yrs**	
≤5	218,136 (83.8)
6–12	28,606 (11.0)
13–19	13,693 (5.2)
**Sex**	
Male	141,301 (54.3)
Female	117,872 (45.2)
Unknown	1,262 (0.5)
**Reason for ingestion**	
Unintentional	245,596 (94.3)
Intentional	13,722 (5.3)
Other	1,117 (0.4)
**Exposure site**	
Residence	257,761 (99.0)
School	561 (0.2)
Other	2,113 (0.8)
Clinical effects*	
CNS	37,164 (81.4)
Gastrointestinal	4,655 (10.2)
Cardiovascular	1,147 (2.5)
Metabolic	346 (0.8)
Other	2,335 (5.1)
**Outcome**	
No effect^†^	78,423 (30.1)
Minor effect^§^	176,435 (67.8)
More serious outcomes^¶^	3,211 (1.2)
Death**	2
Other^††^	2,366 (0.9)
**Management site**	
Managed on-site (non-HCF)	230,032 (88.3)
Managed at HCF	27,795 (10.7)
Unknown	2,608 (1.0)
**Disposition of patients managed at HCF** **(n = 27,795)**	
Hospitalized	4,097 (14.7)
ICU	287 (1.0)
Treated and released	19,892 (71.6)
Other	3,806 (13.7)

**Abbreviations**: CNS = central nervous system; HCF = health care facility; ICU = intensive care unit; RCF = relative contribution to fatality.

* Number of clinical effects (n = 45,647) is greater than the number of symptomatic ingestions (n = 40,665), as some children had more than one symptom.

^†^ No signs or symptoms.

^§^ Minimally bothersome symptoms, self-limited, and resolved without intervention (e.g., self-limited gastrointestinal symptoms).

^¶^ More serious outcomes included moderate effect (systemic symptoms requiring intervention; not life-threatening [e.g., brief seizure readily resolved with treatment, or high fever]), major effect (life-threatening symptoms [e.g., status epilepticus or respiratory failure requiring intubation]), and death.

** RCF: Unknown. A case is classified as RCF "unknown" in the National Poison Data System if the Clinical Case Evidence is not sufficient to rule in or rule out the exposure as the cause of death.

^††^ Cases confirmed as nonexposures and exposures deemed not responsible for the effect.

On page 727, in Figure 2, the y-axis was incorrectly formatted to demonstrate stacked values. Figure 2 has been updated accordingly.

**FIGURE 2:**
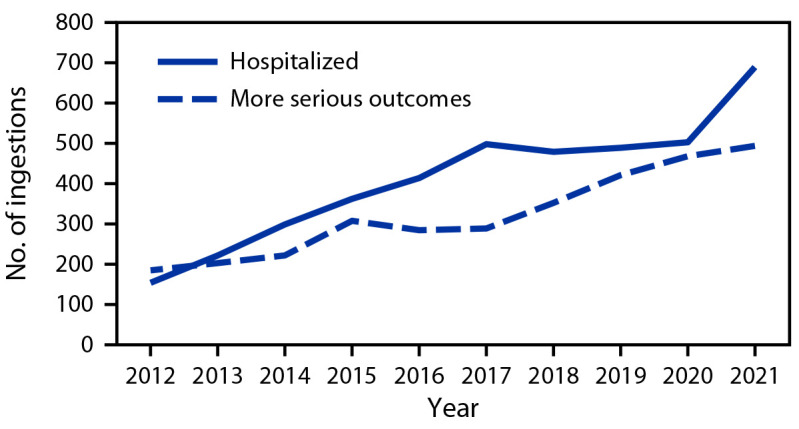
Number of pediatric* melatonin ingestions reported† to poison control centers, by outcome and year — United States, 2012–2021 * Aged ≤19 years. ^†^ More serious outcomes include moderate or major effect or death, as defined by the National Poison Data System Coding Manual. Disposition (including hospitalization) and medical outcome (including more serious outcomes) are not mutually exclusive because persons with more serious outcomes are likely to be hospitalized.

